# A Multi-Frequency Tomographic Inverse Scattering Using Beam Basis Functions

**DOI:** 10.3390/s22041684

**Published:** 2022-02-21

**Authors:** Ram Tuvi

**Affiliations:** John A. and Katherine G. Jackson School of Geosciences, Institute for Geophysics, The University of Texas at Austin, Austin, TX 78758, USA; ram@ig.utexas.edu

**Keywords:** inverse scattering, imaging, wave propagation, beam summation methods

## Abstract

We present an overview of a beam-based approach to ultra-wide band (UWB) tomographic inverse scattering, where beam-waves are used for local data-processing and local imaging, as an alternative to the conventional plane-wave and Green’s function approaches. Specifically, the method utilizes a phase–space set of iso-diffracting beam-waves that emerge from a discrete set of points and directions in the source domain. It is shown that with a proper choice of parameters, this set constitutes *a frame* (an overcomplete generalization of a basis), termed “beam frame”, over the entire propagation domain. An important feature of these beam frames is that they need to be calculated once and then used for all frequencies, hence the method can be implemented either in the multi-frequency domain (FD), or directly in the time domain (TD). The algorithm consists of two phases: in the processing phase, the scattering data is transformed to the beam domain using windowed phase–space transformations, while in the imaging phase, the beams are backpropagated to the target domain to form the image. The beam-domain data is not only localized and compressed, but it is also physically related to the local Radon transform (RT) of the scatterer via a local Snell’s reflection of the beam-waves. This expresses the imaging as an inverse local RT that can be applied to any local domain of interest (DoI). In previous publications, the emphasis has been set on TD data processing using a special class of localized space–time beam-waves (wave-packets). The goal of the present paper is to present the imaging scheme in the UWB FD, utilizing simpler Fourier-based data-processing tools in the space and time domains.

## 1. Introduction

Inverse scattering deals with determining the shape and the composition of an unknown object from measurements of the scattering field data due to a known illumination. This area has a wide range of medical, geophysical, oceanographical, industrial, etc., applications, using electromagnetic, acoustic, elastic, or seismic waves [1,2,3,4,5]. Inverse scattering problems are, in general, non-linear and highly ill-posed, hence accurate solutions typically require iterative schemes and are limited to rather small configurations in the order of wavelengths. For large domains, practical algorithms rely on linearized weak scattering formulations using the Born, Rytov, Physical optics, or other single scattering approximations [2,5] which linearize the relation between the target and the field and provide the basis for diffraction tomography (DT) reconstruction [6].

Inverse scattering requires diversity and relies on the wave data in hand. Depending on the application, it may involve multiple excitation frequencies (or short-pulse response) and/or several illumination directions. With the overall complexity of the problem, full utilization of the wave data is essential to formulate an efficient, robust, and accurate algorithm.

Beam summation (BS) methods are when the wave field is expressed as a superposition of collimated beam waves. Here, we use the generic term “beam” for both the FD formulations where the propagators are Gaussian beams, and for the TD formulations where the propagators are pulsed beams. This provides the proper physical basis for such robust DT reconstruction. Like the plane-wave (PW) spectrum approach, the BS provides a complete spectral basis which is required for DT reconstruction, yet unlike plane-waves, the beam waves provide spatial resolution and they can easily and efficiently be propagated in inhomogeneous media along ray trajectories. Unlike rays, on the other hand, they provide a uniform spectral basis and are insensitive to geometrical optics transition regions. Thus, beams provide a way to convert the wave problem to a ray-based skeleton.

BS methods can be classified into two classes (see review in [7,8]). The first class addresses radiation from localized (point) sources by expressing the field as an angular superposition of collimated beams that emerge radially from the source. This formulation has been derived asymptotically [9,10], but later on it was formulated as an exact spectral identity using complex source beams [11,12] and was also extended to the time domain (TD) [12]. Consequently, it has been used in various applications of propagation, scattering, and inverse scattering.

The other class addresses radiation from extended (aperture) sources, where the field is expressed as a sum of beam propagators that emerge from a discrete phase–space lattice of points and directions in the aperture. These formulations utilize local window (e.g., Gaussian) functions to transform the data to the beam domain, and then propagate the data using beams. These formulations are utilized in the present work where we analyze the data on the measurement aperture and then backpropagate it to the scatterer domain. Early implementation of this approach was based on a Gabor series expansion of the planar field [13,14,15,16] and therefore suffered from two major drawbacks: (a) The Gabor expansion coefficients (the beam amplitudes) are notoriously non-local and unstable, and (b) the beam lattice is frequency-dependent, hence a new lattice should be calculated at each frequency. Both difficulties have been mitigated in the ultra-wide band phase–space beam summation (UWB-PS-BS) method [17,18] which utilizes the overcomplete windowed Fourier transform (WFT) frames. In linear algebra, a frame of an inner product space is a generalization of a basis of a vector space to overcomplete sets. In signal processing, frames provide a redundant, stable way of representing a signal, instead of the Gabor series. The formulation is structured upon a *frequency independent* lattice of beams that emerge from a discrete phase–space lattice of points and directions in the aperture, and utilizes iso-diffracting Gaussian beams (ID-GB) whose propagation parameters are *frequency independent.* These properties make this representation efficient for wideband applications and also allow an extension of the theory to the time-domain (TD) [19].

A major step forward has been the proof in [20] that these phase–space sets of beams constitute a frame not only in the aperture domains but actually everywhere in the propagation domain. This implies that these beam basis functions may be used to expand not only the sources and the field, but also any function of space and in particular the medium inhomogeneities, a property that is being used in our beam-based inverse scattering theory. The theory has been proved originally in the frequency domain (FD) [20] and then extended to the TD in [21].

**Figure 1:**
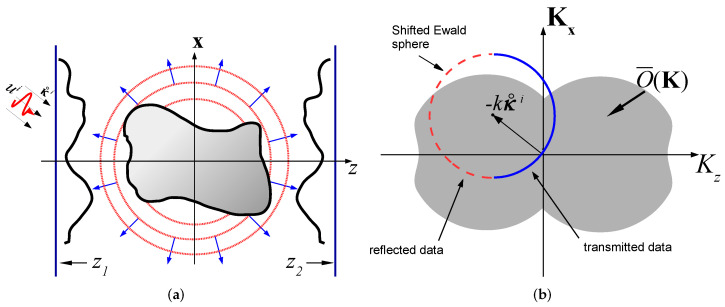
Diffraction tomography and the *K*-space diffraction tomography identity in the spatial and spectral domains. (**a**) The physical configuration. The unknown object O(r) is located between two measurement planes. At $z=z_{1}$ we have an array of sources/receivers, while at $z=z_{2}$ we have an array of receivers. The object is illuminated by the plane-wave (black arrows) of Equation (3) propagating in the direction $\overset{˚}{\kappa }{}^{i}$. In red we plot pulsed plane-wave illumination of Equation (3). The scattered field is measured on the $z_{j}$ planes. (**b**) The DT identity of (8). The plane-wave spectrum of the scattered field $\hat{\tilde{u}}{}_{j}^{s}\left(\xi \right)$ is mapped to values of $\overset{ˉ}{O}(K)$ over a shifted Ewald sphere $K=k(\overset{˚}{\kappa }{}_{j}-\overset{˚}{\kappa }{}^{i})$. The data measured on the j=1,2 planes are illustrated by red dashed and blue solid-line hemispheres, respectively.

As noted above, it has been recognized long ago that BS provides the proper physical basis for a robust DT reconstruction. Examples for point-sources configurations can be found in [22,23,24,25,26,27,28,29,30]. For configuration where the data is measured over a wide aperture (see Figure 1a), it is more suitable to use the extended sources approach noted above: see [31,32,33,34,35,36,37] and [38,39,40,41,42,43,44] for medium reconstruction over a homogenous or inhomogeneous background, respectively. Without going into detailed comparison, the main difference between the methods is in the data representation phase (see [42] for a detailed comparison).

In this work we review the beam-based local inverse scattering theory derived in [36,37], which is based on the frame-based UWB-PS-BS theory discussed above. As noted there, the theory is structured on a frequency-independent phase–space sets of beams that constitute frames everywhere in the propagation domain. This beam frame formulation enables the expansion of both the medium inhomogeneities and the scattering data with the same set of beam-basis functions, thus enabling a direct inversion over the beam domain. In previous publications, the emphasis has been set on TD data processing using special localized space–time beam-waves (wave-packets). This requires somewhat sophisticated mathematics and processing tools. In the present paper, on the other hand, we utilize a simpler FD Fourier-based data-processing approach followed by an integration over the relevant frequency band. The paper makes extensive references to equations or figures in [36,37]. Therefore, to simplify the presentation, we refer to them by the prefixes I and II, respectively.

The advantages of our beam-based DT over the conventional DT approach are:

*a*.  Data localization: The phase–space processing of the scattered data extract the local direction of arrival. The phase space representation of the data is therefore localized along well defined trajectories corresponding to the local direction and time of arrival from the relevant regions in the target domain.
*b*.  Under the Born approximation, the beam-wave scattering mechanism by the medium inhomogeneities is described by local Snell’s reflections from the local stratification, which is related to the local Radon transform (LRT) of the medium inhomogeneities (Section 5 in [36]).
*c*.  As follows from the discussion in items *a* and *b*, the phase space data is directly related to the LRT of the medium inhomogeneities about a given region.
*d*.  The beam-based imaging enables local imaging within a given domain of interest (DoI) by considering only the data that correspond to beams that pass through or near the DoI. This not only reduces the problem complexity, but also reduces the noise level, since data and noise arriving from other regions are a priori filtered out.
*e*.  The beam-based imaging enables backpropagation and imaging over a non-homogeneous background.

The presentation below starts in Section 2 with a review of the main concepts in DT. We proceed in Section 3 by reviewing elements of the beam representation, and in particular of the UWB-PS-BS and the BF concept. The beam-based DT is presented in Section 4, where, as discussed above, we emphasize the multi-frequency data processing as opposed to the more complicated TD processing used in [36,37]. The presentation ends in Section 5 with a practical description of the algorithm, including the choice of the various parameters and numerical examples.

## 2. UWB Diffraction Tomography in the Spectral Plane-Wave Domain: A Review

This section reviews the conventional plane-wave DT algorithms. Referring to the configuration in Figure 1a, we consider the two alternative schemes: The *angular diversity* scheme where the data is measured for several illumination directions $\overset{˚}{\kappa }{}^{i}$ at a given frequency (Section 2.3), and the *frequency diversity* scheme where the data is measured over a wide frequency band for a single illumination direction $\overset{˚}{\kappa }{}^{i}$ (Section 2.4). The frequency diversity scheme may also be performed using a short-pulse illumination and calculated directly in the TD [45] (see also I– Section 3-B in [36]).

### 2.1. Problem Description—Physical Configuration

The physical configuration is illustrated in Figure 1a, where the object is located between two measurement planes, at $z=z_{1}<0$ and at $z=z_{2}>0$. We assume a 3D coordinate frame r=(x,z) where the z-coordinate is normal to the measurement planes, and $x=(x_{1};x_{2})$ are the transversal coordinates. The data is collected over a wide frequency band $\Omega \in [\omega_{\min},\;\omega_{\max}]$. The theory is presented here in the FD, but we also discuss the TD formulation for completeness and clearer interpretation. Field constituents in these domains are related via the temporal Fourier transform

(1) $$\hat{u}(\omega )=\int dt\;u(t)e^{i\omega t},$$

where FD constituents are tagged by an over-hat ˆ.

The unknown object is embedded in a uniform background wavespeed $v_{0}$ and assumed to be lossless and non-dispersive. It is described by the unknown wavespeed v(r), and we define the “object function”

(2) $$O(r)=(v_{0}/v(r){)}^{2}-1$$

(see Equation (7)).

The scattering data may be collected as a function of frequency using time-harmonic plane-wave excitation, or directly in the TD utilizing short-pulse plane-wave. These excitations are given by

(3) $$\hat{u}{}^{i}(r,\omega )=\hat{F}(\omega )e^{ik{\overset{˚}{\kappa }}^{i}\cdot r},\;\;u^{i}(r,t)=F(t-v_{0}^{-1}{\overset{˚}{\kappa }}^{i}\cdot r),$$

where $\hat{F}(\omega )$ is the source spectrum and $k=\omega /v_{0}$ is the wavenumber. The incident wave propagates in the direction

(4) $${\overset{˚}{\kappa }}^{i}=(\xi^{i},\zeta^{i})=\sin\theta^{i}\;\cos\phi^{i}{\overset{˚}{x}}_{1}+\sin\theta^{i}\;\sin\phi^{i}{\overset{˚}{x}}_{2}+\cos\theta^{i}\overset{˚}{z},$$

with $\left(\theta^{i},\phi^{i}\right)$ being the polar angles with respect to the *z* axis, and over-circles denote unit vectors.

The scattered fields measured over the $z_{j}$ planes, j=1,2 are denoted as ${\hat{u}}_{j}^{s}\left(x,\omega \right)$ (see Figure 1a). The PW spectral representation of ${\hat{u}}_{j}^{s}\left(r\right)$ is defined via

(5) u˜ˆjs(ξ)=e±ikζzj∫d2xuˆjs(x)e−ikξ·x,  ζ=1−ξ·ξ,  Imζ≥0.

where lower and upper signs correspond to j=1 and 2, respectfully. We added the $e^{\pm ik\zeta z_{j}}$ phase term in (5) in order to normalize the spectral PWs to the z=0 plane instead of $z=z_{j}$ planes.

Note that we use here the frequency normalized spectral coordinates $\xi =K_{x}/k$ which are related to the PW direction via $\xi =\left(\xi_{1},\xi_{2}\right)=\sin\theta \left(\cos\phi ,\sin\phi \right)$, where (θ,ϕ) are the conventional spherical angles with respect to the *z*-axis so that the scattered PWs propagate in the unit vector direction

(6) $${\overset{˚}{\kappa }}_{j}=(\xi ,\mp \zeta )=(\sin\theta \cos\phi ,\sin\theta \sin\phi ,\cos\theta ),\;\;j=1,2.$$

The spectral ranges |ξ|<1 and |ξ|>1 define the *propagation spectrum* and *evanescent spectrum*, respectively. Typically, DT formulations are restricted only to the propagation spectrum data (see discussion after Equation (9)).

### 2.2. The DT Identity

According to the weak scattering (first Born) approximation of the Lippmann–Schwinger integral equation, the scattered field can be expressed as [5]

(7) $${\hat{u}}^{s}(r)=k^{2}\int_{V}d^{3}r^{\prime }{\hat{u}}^{i}(r^{\prime })O(r^{\prime })\hat{G}(r,r^{\prime }),$$

where $\hat{G}=\frac{e^{ik|r-r^{\prime }|}}{4\pi |r-r^{\prime }|}$ is the 3D Green’s function in the uniform background. This approximation is valid if O(r)≪1 and in addition $kLO_{\max}<1$, where *L* is the spatial support of *O* and $O_{\max}$ is its maximal value.

Inserting (7) into (5) and using the spectral representation of $\hat{G}$, we obtain (I-7),

(8) $$\hat{\tilde{u}}{}_{j}^{s}(\xi )≃\frac{k}{-2i\zeta }\bar{O}(K){|}_{K=k({\overset{˚}{\kappa }}_{j}-{\overset{˚}{\kappa }}^{i})}\;\;\;\;|\xi |<1,$$

where ${\overset{˚}{\kappa }}_{j}$ and ${\overset{˚}{\kappa }}^{i}$ are given by (6) and (4), and

(9) $$\bar{O}(K)=\int d^{3}rO(r)e^{-iK\cdot r},\;\;\;\;K=(K_{1},K_{2},K_{z}),$$

is the *K*-space distribution of O(r). Equation (8) is referred to as the *DT identity*. It relates the scattering data in the ${\overset{˚}{\kappa }}_{j}$ directions to values of $\bar{O}(K)$ at the points $K=k({\overset{˚}{\kappa }}_{j}-{\overset{˚}{\kappa }}^{i})$. As illustrated in Figure 1b, these points define a *K*-space sphere with radius *k* that is centered at $K=-k{\overset{˚}{\kappa }}^{i}$, which is referred to as the *shifted Ewald sphere*. Note from (6) that the left and right hemispheres (plotted as red and blue, respectively) correspond to data from the $z_{j}$ measurement plane with j=1,2, respectively.

The DT identity above applies only to the propagation spectrum |ξ|<1. Adding the evanescent spectrum may improve the resolution. However, the contribution of the evanescent spectrum is exponentially weak and hence has a low signal to noise ratio. In addition, backpropagating this data to form the image amplifies the noise level exponentially. For these reasons, the evanescent spectrum contribution is usually neglected except for near field imaging schemes.

In view of the DT identity, one may obtain a full K-space coverage of the object function by measuring the scattering response for several illumination directions or several frequencies [2,5]. These alternative schemes are reviewed in the following sections.

### 2.3. Object Reconstruction via Angular Diversity

**Figure 2:**
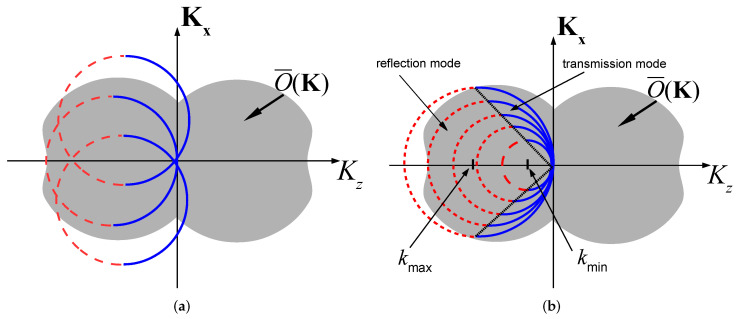
*K*-space reconstruction. (**a**) Angular diversity reconstruction: Changing the direction of illumination $\overset{˚}{\kappa }{}^{i}$ and measuring the transmitted data only provides a coverage of the K-space within a sphere of radius $\sqrt{2}k$ [5]. (**b**) Frequency diversity reconstruction: Changing the excitation frequency for a single illumination direction $\overset{˚}{\kappa }{}^{i}$ provides coverage of the K-space as indicated. The figure is plotted for $\overset{˚}{\kappa }{}^{i}=\overset{˚}{z}$.

The angular diversity approach is illustrated in Figure 2a. Changing the illumination directions ${\overset{˚}{\kappa }}^{i}$ while keeping the operational frequency *k* constant changes the centers of the shifted Ewald sphere and provides a different coverage of the *K* space. Aggregating the response for several illumination directions recovers $\bar{O}\left(K\right)$. Note that for lossless (real) objects, $\bar{O}\left(K\right)={\bar{O}}^{*}\left(-K\right)$, so that only half of the K-space needs to be recovered.

As one observes from Figure 2a, the transmitted data on $z_{2}$ recovers the *K*-space distribution of the object in a sphere of radius $\sqrt{2}k$ about the origin. Thus, the object may be recovered using only transmitted data, as long as *k* is chosen to be large enough to provide full coverage of $\bar{O}(K)$. Note that in the limit of k→∞, the transmitted data hemispheres in Figure 2a reduce to planar surfaces normal to ${\overset{˚}{\kappa }}^{i}$ that pass through the origin, thus providing the *K*-space representation of conventional X-ray tomography [46].

One option to reconstruct O(r) is to recover $\bar{O}(K)$ and then apply the inverse Fourier transform of (9). This approach requires interpolation of the data from the shifted Ewald spheres to a Cartesian *K*-domain grid [47], and therefore requires a large number of illuminations.

The “filtered backpropagation” reconstruction algorithm [6,47] overcomes this difficulty by circumventing the need to recover $\bar{O}(K)$ and operating, instead, directly on the scattering data. In this approach, the scattering data is multiplied by a spectral filter and then back-propagated to the object domain. This reconstruction approach is analogous to the X-ray tomography filtered backprojection algorithm of [48], where the filtered data is back-projected along straight lines.

### 2.4. Object Reconstruction via Frequency Diversity (UWB Tomography)

In the frequency diversity approach, the data is collected over a wide frequency spectrum $\Omega \in [\omega_{\min},\;\omega_{\max}]$ for a fixed illumination direction. This can be done either in a frequency by frequency approach or by using a short-pulse illumination. As noted earlier, in this paper we emphasize the multi-frequency approach. The readers are referred to I–Section 3-B in [36] for the TD formulation, which has an important cogent physical interpretation, but is not utilized here.

As illustrated in Figure 2b for illumination along the positive *z* axis, changing the illumination frequency changes the radius of the shifted Ewald sphere. One observes that the reflected data recovers the *K*-space distribution of *O* inside a π/4 cone with an axis along the negative $K_{z}$ axis and base radii between $k_{\min}$ and $k_{\max}$, while the transmitted data recovers the complementary *K*-space part. As noted before, only half of the *K*-space is needed to recover the real function *O*. Otherwise, several illumination directions are required. More illuminations also add robustness.

As follows from Figure 2b, the reflection data on the $z_{1}$ plane mainly recovers the object variations along the *z* axis, while the transmitted data on the $z_{2}$ plane recovers the transversal variation (see also Snell’s law interpretation in I-Section 3-B in [36]. Thus, for quasi-stratified media with weak transversal variations, it may be sufficient to measure only the reflected data on the $z_{1}$ plane, but may not be sufficient for objects with a large transversal variations. Another limitation is the missing data for $|K_{z}|<2k_{\min}$ (see Figure 2b), while the missing data for $|K_{z}|>2k_{\max}$ can be measured by using higher frequencies. As follows from Figure 2b, several illumination directions may increase the transversal resolution and also add data at small |K|. Note also that the maximal axial resolution for the case of normal incidence is $\delta_{z}=\pi /k_{\max}$.

The object can be reconstructed using an inverse transform of $\bar{O}(K)$. However, for the same reasons discussed in Section 2.3, a filtered backpropagation approach is preferable. Backpropagation can be calculated in several alternative ways. For simplicity, we present here the spectral integration approach. Given the scattering data ${\hat{u}}^{s}\left(x,\omega \right)$ over the $z_{j}$ planes, the backpropagated fields into the $z>z_{1}$ and $z<z_{2}$ regions are given by (see (5))

(10) $$\hat{u}{}_{j}^{b}(r)=(\frac{k}{2\pi }{)}^{2}\int_{|\xi |<1}d^{2}\xi \hat{\tilde{u}}{}_{j}^{s}(\xi )e^{ik(\xi \cdot x\mp \zeta z)},$$

where we restrict the integration to the visible spectrum |ξ|<1.

The “imaging field,” or the “filtered backpropagated field” corresponding to the data on the j=1,2 plane is given by (II-2)

(11) $$\hat{I}{}_{j}(r,\omega )=v_{0}^{-1}k^{-2}{\overset{˚}{\kappa }}^{i}\cdot \nabla [e^{-ik{\overset{˚}{\kappa }}^{i}\cdot r}\hat{u}{}_{j}^{b}(r,\omega )].$$

The corresponding “partial images” are obtained by summing over the relevant frequency band (II-3)

(12) O˘(r)=2Re1π∫0∞dω Iˆj(r,ω).

If the data is given on both planes, then the “complete image” is given by

(13) $$\overset{˘}{O}(r)={\overset{˘}{O}}_{1}(r)+{\overset{˘}{O}}_{2}(r).$$

The features of O(r) that are described by ${\overset{˘}{O}}_{j}$ have been discussed above in connection with Figure 2b. As noted there, in many situations it is sufficient to recover only ${\overset{˘}{O}}_{1}$.

The derivation of the filtered backpropagation imaging algorithm in (10)–(12) is done by inserting the Born approximated data of (8) into (10).

## 3. Mathematical Background on the UWB-PS-BS

### 3.1. The Windowed Fourier Transform (WFT) Frame Representation of the Field

As noted in the introduction above, the phase–space beam summation representation is based on the theory of WFT frame expansion of the field. Following [17], the theory is presented here in the context of radiation into the half-space z>0 in a 3D coordinate space r=(x,z), $x=(x_{1},x_{2})$, due to a time harmonic field ${\hat{u}}_{0}\left(x,\omega \right)$ defined over the plane z=0.

The WFT frame set $\left\{{\hat{\psi }}_{\mu }\left(x,\omega \right)\right\}$ is defined by (Equation (22)] [17])

(14) $${\hat{\psi }}_{\mu }(x,\omega )=\hat{\psi }(x-m\bar{x})e^{ikn\bar{\xi }\cdot (x-m\bar{x})},$$

with $\hat{\psi }$ being a localized window function (typically a Gaussian, see more details below) and $\mu =\left(m,n\right)$ being a 4-index. The frame elements are localized about the spatial (x) and spectral (ξ) phase space lattice

(15) $$(x_{m},\;\xi_{n})=(m\bar{x},\;n\bar{\xi })=(m_{1}\bar{x},m_{2}\bar{x};\;n_{1}\bar{\xi },n_{2}\bar{\xi }),$$

where $\left(\bar{x},\bar{\xi }\right)$ defines the lattice unit cell. As will be shown, the points $\left(x_{m},\;\xi_{n}\right)$ define the beams’ initiation points and propagation directions (see Equation (21) below). To constitute a frame, the set above needs to fully cover the phase space, i.e., the unit cell area should be less than 2π, implying that

(16) $$k\bar{x}\bar{\xi }=2\pi \nu ,$$

with ν<1 being the overcompleteness parameter and the limit ν=1 define the critically complete limit. As will be shown in Section 3.2 below, the frame over completeness provides a local and stable representation of the field (Equation (13) [17]), with it also being used to derive an UWB representation of the field (see Equations (Equations (33)–(35) [17])).

The WFT frame can be used to expand ${\hat{u}}_{0}\left(x\right)$ in the form

(17) $${\hat{u}}_{0}(x)=\sum_{\mu }{\hat{a}}_{\mu }{\hat{\psi }}_{\mu }(x).$$

In view of the overcompleteness, the coefficients set $\left\{{\hat{a}}_{\mu }\right\}$ is not unique. A particularly convenient set with a minimum $\ell_{2}$ norm is obtained by using the dual frame $\left\{{\hat{\varphi }}_{\mu }(x)\right\}$ which has the same structure as $\left\{{\hat{\psi }}_{\mu }\right\}$ in (15) except that the mother window $\hat{\psi }(x)$ is replaced by the dual mother window $\hat{\varphi }(x)$. The resulting canonical coefficient set is given by (Equation (23) [17]).

(18) $${\hat{a}}_{\mu }=\langle {\hat{u}}_{0}(x),{\hat{\varphi }}_{\mu }(x)\rangle$$

where $\langle f,g\rangle =\int fg^{*}$ is the conventional $L_{2}$ inner product in the transverse coordinate **x**. The canonical coefficients ${\hat{a}}_{\mu }$ in (18) are readily identified as the local spectrum of ${\hat{u}}_{0}\left(x\right)$ windowed with respect to ${\hat{\varphi }}_{\mu }$ about the phase–space points $\left(x_{m},\xi_{n}\right)$.

Generally, $\hat{\varphi }$ should be calculated numerically, for a given $\hat{\psi }$ and lattice $\left(\bar{x},\bar{\xi }\right)$. However, if the lattice is sufficiently overcomplete, (ν≲1/3) $\hat{\varphi }\propto \hat{\psi }$ can be approximated by (Equation (11) [17])

(19) $$\hat{\varphi }(x)\approx \nu^{2}\hat{\psi }(x)/\|\psi {\|}^{2}.$$

There are mainly two reasons to prefer the use of this highly overcomplete parameter regime, even though it implies a larger number of terms in the phase–space expansion (17): (i)—as follows from (19), in this parameter regime $\hat{\varphi }$ is localized both spatially and spectrally, so that the expansion (17) comprises local and stable coefficients. (ii)—$\hat{\varphi }$ is given analytically via (19) and does not have to be to calculated numerically. Reason (ii) is critical for UWB problems where $\hat{\varphi }$ needs to be found for each ω.

The radiated field in z>0 is obtained now by replacing ${\hat{\psi }}_{\mu }\left(x\right)$ in Equation (17) by beam propagators (Equation (24) [17])

(20) $$\hat{u}(r)=\sum_{\mu }{\hat{a}}_{\mu }{\hat{\Psi }}_{\mu }^{+}(r),$$

(21) $${\hat{\Psi }}_{\mu }^{+}(r)=(\frac{k}{2\pi }{)}^{2}\int d^{2}\xi \hat{\tilde{\psi }}{}_{\mu }(\xi )e^{ik(\xi \cdot x+\zeta z)}.$$

${\hat{\Psi }}_{\mu }^{+}(r)$ are identified as the fields that are radiated forward into z>0 by ${\hat{\psi }}_{\mu }\left(x\right)$. In (21), $\hat{\tilde{\psi }}{}_{\mu }(\xi )=\hat{\tilde{\psi }}\left(\xi -\xi_{n}\right)e^{-ik\xi \cdot x_{m}}$ is the PW spectrum (5) of ${\hat{\psi }}_{\mu }\left(x\right)$, with $\hat{\tilde{\psi }}(\xi )$ being the spectrum of the “mother window” $\hat{\psi }\left(x\right)$. If $\hat{\psi }\left(x\right)$ is wide on a wavelength scale, then ${\hat{\Psi }}_{\mu }^{+}(r)$ behave like collimated beams, emerging from the points $x_{m}$ over the z=0 plane and directions ${\overset{˚}{\kappa }}_{n}=\left(\xi_{n},\zeta_{n}\right)=\left(\sin\theta_{n}\cos\phi_{n},\sin\theta_{n}\sin\phi_{n},\cos\theta_{n}\right)$ with $\zeta_{n}=\sqrt{1-|\xi_{n}{|}^{2}}$.

### 3.2. UWB Considerations

In general, the applications in hand require UWB excitations. Following [17], we use the following frequency-scaling of the WFT frame set that renders the theory amenable for UWB field representations:

*(1) Frequency independent beam skeleton:* As implied from Equation (16) above, the beam lattice should be recalculated for each frequency. For efficient UWB representations, it is required to have the same beam lattice $\left(x_{m},\xi_{n}\right)$ over the entire frequency band. In view of (16), this requirement implies (Equation (10) [21])

(22) $$\nu (\omega )=\nu_{\max}\frac{\omega }{\omega_{\max}},\;\;\;\;\omega \in [\omega_{\min},\omega_{\max}],$$

with $\nu_{\max}$ being the value of ν at $\omega_{\max}$, so that $\nu <\nu_{\max}$ for all $\omega <\omega_{\max}$. Typically, we use $\nu_{\max}=1/3$ (see discussion in (25) below).

*(2) Iso-diffracting propagators:* We use iso-diffracting (ID) Gaussian windows which are scaled with frequency in the form (Equation (27) [17])

(23) $${\hat{\psi }}_{\mathrm{ID}}(x)=e^{-k|x{|}^{2}/2b},\;\;\;\;k<0,$$

where b>0 is a frequency independent parameter. Inserting (23) into (21) and evaluating the integral one finds that the resulting propagators are ID-GBs, with *b* being the collimation distance. The ID designation of these Gaussian beams is due to the fact that their collimation distance *b* is frequency independent. This property implies that the beam propagation parameters are frequency independent even in inhomogeneous medium. Furthermore, when transformed into the TD, they give rise to ID-Pulsed beams (ID-PB) which are space time wave-packets that maintain their wave-packet structure even through propagation in inhomogeneous medium [49]. Explicit expressions for the corresponding phase–space beam propagators of (26) in free space are given in (Equations (28)–(29)) [17].

Typically *b* is chosen by the molder and depends on the application (see discussion in the numerical example below), but also should satisfy the condition $k_{\min}b\gg 1$, which implies that the beams are highly collimated over the entire frequency band.

*(3) Snug frame:* The frame is optimal (or snug) when the frame elements are matched to the phase–space lattice $\left(\bar{x},\bar{\xi }\right)$ (in the sense that they should provide a balanced phase–space coverage). This requirement implies the relation $b=\bar{x}/\bar{\xi }$ [17]. Combining this condition with (16) one obtains (Equation (A2) [21]),

(24) $$(\bar{x},\bar{\xi })=\sqrt{\frac{2\pi \nu_{\max}}{k_{\max}}}(b^{1/2},\;{-b}^{1/2}).$$

*(4) Simple expression for the dual frame function:* In view of (19) we have for $\nu_{\max}=1/3$ (Equation (A3)[21]),

(25) $$\hat{\varphi }{}_{\mathrm{ID}}(x)≃\frac{\nu_{\max}^{2}}{\pi bk_{\max}^{2}}k^{3}\hat{\psi }{}_{\mathrm{ID}}(x),\;\;\;\;\omega <\omega_{\max}.$$

Over this regime $\hat{\varphi }$ is spatially and spectrally localized, and leads to a stable and localized expansion coefficients [17].

The properties above yield an efficient multi-frequency representation where the phase–space lattice and propagation parameters should be calculated only once for all frequencies in the band. These advantages also allow a simple transformation of the beam representation to the TD [19,21].

### 3.3. The Beam Frame Theorem

**Figure 3:**
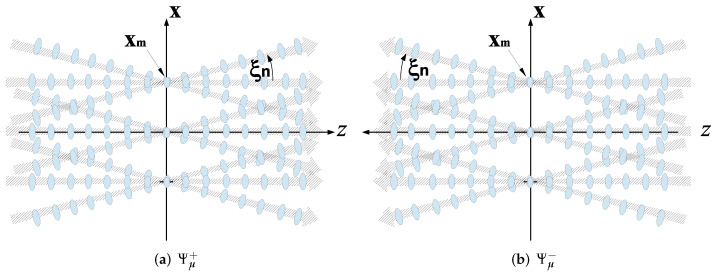
The forward/backward propagating beam frames $\Psi_{\mu }^{\pm }$. (**a**) The forward and (**b**) the backward beam frames $\Psi_{\mu }^{\pm }$. The BFs are illustrated by the hatched arrows. The ellipses correspond to the pulsed-beam-frames that are used in the TD formulations and are not considered here (see [21]).

Following (21), we define the set of forward and backward propagators $\left\{{\hat{\Psi }}_{\mu }^{\pm }(r)\right\}$ (compare Equation (21))

(26) $${\hat{\Psi }}_{\mu }^{\pm }(r)=(\frac{k}{2\pi }{)}^{2}\int_{|\xi |<1}d^{2}\xi \hat{\tilde{\psi }}{}_{\mu }(\xi )e^{ik(\xi \cdot x\pm \zeta z)},\;\;\;\;|\xi_{n}|\leq \xi_{0}<1.$$

where the parameter $\xi_{0}$ is typically chosen close to 1. Note that this subset includes only “propagating beams” whose spectrum, which is localized around $\xi_{n}$, is located in the propagating spectrum range |ξ|<1. We denote this subset by the index $\mu_{P}$. Inserting the ID Gaussian windows of (23) into (26) and evaluating the integrals asymptotically one readily identifies ${\hat{\Psi }}_{\mu }^{\pm }$ as forward and backward ID-GB that propagate from z=∓∞ to ±∞ in the directions $\overset{˚}{\kappa }{}_{n}^{\pm }=\left(\xi_{n},\pm \zeta_{n}\right)=\left(\sin\theta_{n}\cos\phi_{n},\sin\theta_{n}\sin\phi_{n},\cos\theta_{n}\right)$ (see Figure 3), while for z=0 they converge to the PS lattice of Section 3.1 as illustrated in Figure 3.

As has been established by **the beam frame theorem** in [20], the beam-sets ${\left\{{\hat{\Psi }}_{\mu }^{\pm }\left(r\right)\right\}}_{\mu_{P}}$ constitute frames (hence referred to as “beam frames” (BF)) at any z=const. plane over the Hilbert space $H_{P}$ of functions whose spectrum is bounded in the propagation domain $|\xi |<\xi_{0}$, with the set $\left\{{\hat{\Phi }}_{\mu }^{\pm }\left(r\right)\right\}$ being the dual frames. The propagators ${\hat{\Phi }}_{\mu }^{\pm }$ have the same form as ${\hat{\Psi }}_{\mu }^{\pm }$ in (26), except that ${\hat{\tilde{\psi }}}_{\mu }$ are now replaced by ${\hat{\tilde{\varphi }}}_{\mu }$. Note that in view of (25), ${\hat{\Phi }}_{\mu }^{\pm }$ are proportional to ${\hat{\Psi }}_{\mu }^{\pm }$.

It follows from the beam frame theorem that any function over $H_{P}$ may be expanded by the BF. This observation is very important in the context of inverse scattering since it implies that both the scattered field and the medium are expanded on the same basis.

An important special case of the above is when the BF are used to expand forward or backward propagating wave-fields ${\hat{u}}^{\pm }\left(r\right)$. In view of the theorem, $u^{+}$ may be expanded using ${\hat{\Psi }}_{\mu }^{+}$, and $u^{-}$ may be expanded using ${\hat{\Psi }}_{\mu }^{-}$, but the physically meaningful choice is to expand $u^{\pm }$ using ${\hat{\Psi }}_{\mu }^{\pm }$, respectively, viz (Equation (32) [20])

(27) $${\hat{u}}^{\pm }(r)=\sum_{\mu \in \mu_{P}}{\hat{A}}_{\mu }^{\pm }{\hat{\Psi }}_{\mu }^{\pm }(r),$$

where the summation includes only “$\mu_{P}$ propagating” frame-beams with no evanescent spectrum. As has been established by **the expansion coefficient invariance theorem** in [20], ${\hat{A}}_{\mu }^{\pm }$ may be calculated by projecting ${\hat{u}}^{\pm }\left(r\right)$ on the dual frame ${\hat{\Phi }}_{\mu }^{\pm }(r)$ over any $z=z^{\prime }$ plane, giving the same result, i.e., (Equation (33) [20])

(28) $${\hat{A}}_{\mu }^{\pm }=\langle {\hat{u}}^{\pm }(r),{\hat{\Phi }}_{\mu }^{\pm }(r)\rangle {|}_{z\prime }=\langle {\hat{u}}^{\pm }(x),{\hat{\varphi }}_{\mu }^{\pm }(x)\rangle {|}_{z=0}$$

where the last expression describes the canonical WFT coefficients of (18) evaluated over the z=0 plane.

Finally we note that in [21], the BF theorem has been extended to the TD using ID-PB propagators.

## 4. UWB Beam-Based Diffraction Tomography: Multi-Frequency Formulation

**Figure 4:**
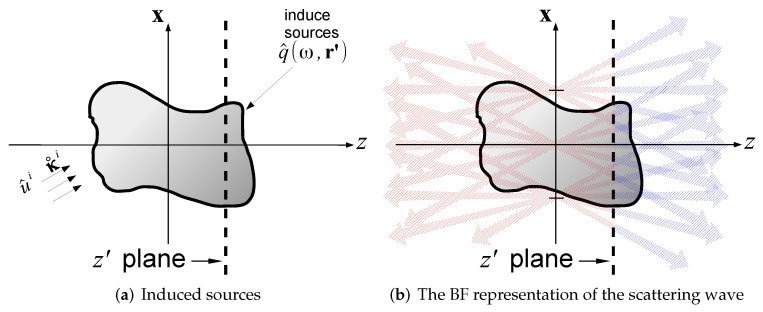
The scattering mechanism within the propagating frame formulation. The incident field that propagates through the medium (see subplot (**a**)) gives rise to induced sources. At each z=const. plane, these sources are expanded by the forward/backward propagating BFs, giving rise to the forward/backward scattered fields depicted in subplot (**b**) in blue and red, respectively.

The beam frame concept provides a framework to formulate the beam-based inverse scattering algorithm. Using the BFs, we may use the same set of beam basis functions to expand both the scattering data and the medium (actually, the sources that are induced due to the medium heterogeneities). As illustrated in Figure 4, the inverse problem is thereby described by the local interaction between the beam amplitudes and the unknown object. As noted in the introduction, optimal localization is obtained in the time-domain formulation, using localized space–time wave-packets. This, however, requires somewhat sophisticated processing tools [21]. In the present section we present only the multi-frequency formulation that utilizes conventional FD data-processing tools followed by integration over all the frequencies. The readers are referred to [36,37] for the TD interpretation.

### 4.1. The Inversion Algorithm

Given the scattering data over the $z_{j}$ planes, the BF representation of the scattering fields into the $z≶z_{j}$ half spaces are given by (see (27))

(29) $${\hat{u}}_{j}^{s}(r,\omega )=\sum_{\mu \in \mu_{P}}{\hat{A}}_{\mu }^{j}(\omega ){\hat{\Psi }}_{\mu }^{\mp }(r,\omega ),\;\;\;\;z≶z_{j},$$

where, as before, upper and lower signs correspond to j=1 and j=2, respectively. The expansion coefficients calculated via (28),

(30) $${\hat{A}}_{\mu }^{j}(\omega )=\langle {\hat{u}}_{j}^{s}(x,\omega ),{\hat{\Phi }}_{\mu }^{\mp }(r,\omega ){|}_{z_{j}}\rangle .$$

Following the discussion after (7), these coefficients extract the local PW spectrum of the scattering data. Note that, as was done in the PW spectrum of Equation (5), the scattering WFT operation normalizes the scattering on the $z_{j}$ planes to their phase centers on the z=0 plane. The coefficients in (30) are referred to as the beam-domain data.

The backpropagated fields ${\hat{u}}_{j}^{b}(r,\omega )$ are obtained by extending (29) as is to $z≷z_{j}$ (see (II-9)). The “imaging fields” are then calculated by inserting (29) into Equation (11). In view of the local structure of the ${\hat{\Psi }}_{\mu }^{\mp }$ propagators, we obtain the explicit expression (II-11)

(31) $$\hat{I}{}_{j}(r)≃\frac{2}{i\omega }e^{-ik{\overset{˚}{\kappa }}^{i}\cdot r}\sum_{\mu }{\hat{A}}_{\mu }^{j}(\omega )\cos^{2}(\frac{\gamma_{n}^{\mp }}{2}){\hat{\Psi }}_{\mu }^{\mp }(r,\omega ),$$

where $\gamma_{n}^{\mp }$ represents the angle between the illumination direction $-{\overset{˚}{\kappa }}^{i}$ and the beam direction ${\overset{˚}{\kappa }}_{\mu }^{j}$ (which actually depends only on *n*. Finally, the reconstructed object is calculated via (12) and (13). For full details, the reader should refer to Appendices II-A,B.

### 4.2. Interpretation within the Born Approximation

In order to gain insight into the structure of the beam-domain data, we insert the Born approximation of the scattered field in (7) into (30). The resulting relation between O(r) and the beam data is given by (I-21)

(32) $${\hat{A}}_{\mu }^{j}(\omega )≃\langle O(r),{\hat{\Lambda }}_{\mu }^{j}(r,\omega )\rangle_{V},\;\;{\hat{\Lambda }}_{\mu }^{j}(r,\omega )=k/2i\cos\theta_{n}e^{-ik{\overset{˚}{\kappa }}^{i}\cdot r}{\hat{\Phi }}_{\mu }^{\mp }(r,\omega ),$$

where the integration covers the entire scatterer domain. Thus, within the Born approximation, the data is described as projections of O(r) on the beam axis, using the projection kernels ${\hat{\Lambda }}_{\mu }^{j}\left(r,\omega \right)$. As shown in I-Section 5-B in [36], this projection extracts the local stratification of *O* along the beam axis at an angle $\gamma_{n}^{\mp }$ defined in (31). This implies that the scattering amplitudes ${\hat{A}}_{\mu }^{j}$ are obtained from Snell’s reflections due the stratification components in O(r) along the μ beam axis, so that strong amplitudes are obtained only for those μ (locations and directions) that correspond to the stratification of O(r) along the μ beam axis. Note that (32) is the local generalization of (7), where the BF basis is used instead of the conventional Green’s function that radiates in all directions.

Further localization along the beam axis is provided by using the TD formulation in (I-27)-(I-32). However, as noted earlier, the TD formulation is not presented here since our goal in this paper is to present the pragmatic and practical formulation in the FD where all the operations are based on Fourier-type integrals. The readers are referred to [36,37] for more details on the TD formulations.

To further explore the FD beam data representation, we consider the spectral representation of ${\hat{A}}_{\mu }^{j}$. Substituting (5) into (30) and changing the order of integrations, ${\hat{A}}_{\mu }^{j}$ can be expressed as (I-22)

(33) $${\hat{A}}_{\mu }^{j}≃(\frac{k}{2\pi }{)}^{2}\int d^{2}\xi \frac{ik}{2\zeta }\hat{\tilde{\varphi }}{}_{\mu }^{*}(\xi )\bar{O}(K){|}_{K=k({\overset{˚}{\kappa }}_{j}-{\overset{˚}{\kappa }}^{i})}.$$

The expression is the local alternative to the plane-wave-based DT identity in (8). It is recognized as ${\overset{˚}{\kappa }}_{n}^{\pm }$ samples of the value of $\bar{O}(K)$ over the shifted Ewald sphere defined in (9). The spectral width of these samples is that of ${\hat{\tilde{\varphi }}}_{\mu }(\xi )$.

## 5. Numerical Examples

In this section, we demonstrate the beam-based DT algorithm through a numerical example. We begin with a step-by-step summary of the algorithm, including guidelines for choosing the parameters.

### 5.1. A Step by Step Summary of the Algorithm

**Phase I—the experimental setup**

**Figure 5:**
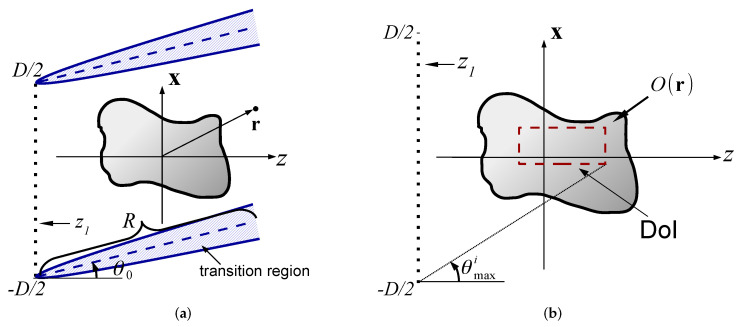
Guidelines for choosing the experimental setup. (**a**) The size of the array should be wide enough to provide a plane-wave illumination at the target range *R*. The scan angle is limited in order to be sufficiently far for the end-point diffraction zone. (**b**) For local imaging, only the part of the object inside the DOI should satisfy the conditions above.

1. We consider a realistic case where the object is illuminated by an array of *M* independent point transducers over the $z_{1}$ plane, as illustrated in Figure 5a. We illustrate here only the reflected data on the $z_{1}$ plane, since in many applications (e.g., geophysics) the transmission field cannot be measures, and in some cases this data is not needed (see discussion in Section 2.4). If, however, the transmitted data at $z_{2}$ is available, then the receiver array considerations are similar.
2. The data is measured by exciting the sources one at a time by short pulse F(t) that spans the desired frequency band $\Omega \in [\omega_{\min},\;\omega_{\max}]$ as needed to obtain the desired K-space coverage. The result is an M×M data matrix $U_{pq}^{s}\left(t\right)$ describing the response at the *p* receiver due to an excitation by the *q* source.
3. The data is sampled at the proper Nyquist rate and then converted to the FD via FFT, giving rise to the data matrix $\hat{U}{}_{pq}^{s}\left(\omega \right)$. Before the calculation, the time-series are padded by zeros to avoid aliasing of the final image when it is generated by integration over all the frequencies. Note that in some applications, $\hat{U}{}_{pq}^{s}\left(\omega \right)$ may be measure directly in the FD.
4. The response to time-harmonic PW excitations at different angles is synthesized from $\hat{U}{}_{pq}^{s}\left(\omega \right)$ by *q*-stacking the array data with proper phase terms. The result provides the PW data to the phase–space beam-based processing.
  One may also calculate the time-harmonic PW spectrum of the scattered field via *p*-stacking with proper phase terms. The result is an M×M data matrix $\hat{\tilde{U}}{}_{p\prime q\prime }^{s}\left(\omega \right)$ describing the $p^{\prime }$ spectral PW due to an excitation by the $q^{\prime }$ incident PW. As noted earlier, before we do the stacking, the array dimensions should be zero- padded to avoid aliasing of the final image when the images are generated by spectral integrations.
5. As illustrated in Figure 5a, the spectral information that can be covered by the array is determined by the size of the array and by the target range *R*. The size of the array should be chosen to provide sufficient spectral coverage of the target. In general, *R* should be within the Fresnel zone of the array, i.e., $R\ll {\left(D\zeta^{i}\right)}^{2}/\lambda$. Note also that due to the finite size of the array, one should avoid the array transition zone illustrated in Figure 5a.
6. The array elements inner spacing should, in general, satisfy the Nyquist sampling rate kd=π. However, since the target range satisfies kR≫1, it is only required that the phase difference between adjacent elements will be small, yielding a sparser array with $d<\sqrt{\frac{\pi }{8k_{\max}}R}$.

**Phase II—Constructing the phase space lattice**

The next step is to set up the phase–space lattice and choose the expansion parameters

1. **Choosing the beam parameter *b*:** As discussed in Section 3.2, the ID Gaussian windows in (23) are fully determined by the parameter *b*. The considerations of choosing *b* were widely studied in [21,36] for the application of local inverse scattering. This parameter balances between the beam collimation length and the beamwidth. We choose *b* to be on the order of the DoI domain so that the beams are collimated throughout the DoI while being small enough for transversal resolution.
2. **The phase–space lattice:** The guidelines for constructing the UWB beam lattice are discussed in Section 3.2. As discussed there, we choose $\nu_{\max}=1/3$ which balances between stable expansion frame and moderate over-completeness (relatively small number of elements). The optimal values for $\left(\bar{x},\bar{\xi }\right)$ are given by (24).
3. **The phase–space propagators:** The frame elements $\left\{{\hat{\Psi }}_{\mu }^{\pm }\left(r\right),\;{\hat{\Phi }}_{\mu }^{\pm }\left(r\right)\right\}$ are calculated via (26). For the Gaussian windows (23), the results are ID-GB propagators (see explicit expressions in (Appendices A,C [21]).
4. **Limited physical data:** We need to consider only beams whose initiation point $x_{m}$ are supported by the array size. The maximal value of $\xi_{n}$ is determined by the scan angle. If, for example, the scan angle is $60^{\circ }$, then $|\xi_{n}|<\frac{\sqrt{3}}{2}$.

**Phase III—Calculating the beam data**

1. **Calculating the expansion coefficients:** The coefficients ${\hat{A}}_{\mu }^{j}$ are calculated via (30).
2. **Filtering out low amplitude data:** As discussed in Section 4.2, the beam data is related to the local Snell’s reflections of the beams by the local stratification in O(r), which in turns are determined by the LRT of O(r). We therefore threshold low amplitude beams at a level of 40 dB.

**Phase IV—Local reconstruction via beam backpropagation**

**Figure 6:**
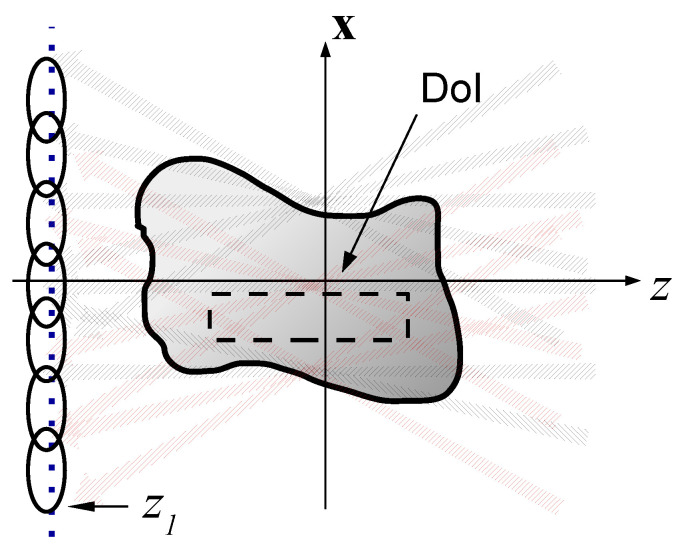
An overview of the local inversion algorithm. The beam expansion of the scattered field is plotted as gray arrows. Only those covering the array are plotted. The scattering data is then transformed to beam amplitudes by stacking the receivers data via (30), as schematized by the black ellipses. Only beams with high amplitude are considered and backpropagated via (31). The image in the DoI (black rectangle) is obtained by aggregating the contribution of beams that pass inside the DoI (red beams).

1. **Beam backpropagation within the DoI:** Next, following Section 4.1, we backpropagate the beams whose amplitudes ${\hat{A}}_{\mu }^{j}$ are larger than the threshold set above. We consider only the beams passing through the DoI (see Figure 6) or no further than 3 beam-widths away from the DoI (this distance is consistent with an effective threshold of 40 dB).
2. **The image:** The imaging fields are calculated via (31), and finally the image is calculated via Equations (12)–(13).

### 5.2. Example A: A Smooth and Quasi Stratified Medium. UWB Reflection Mode Data 

**Figure 7:**
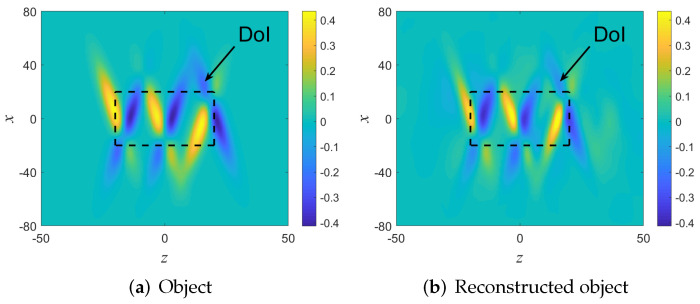
Example A: (**a**) The original (**a**) and the reconstructed (**b**) object functions. The DoI is illustrated by the black rectangle.

The medium is plotted in Figure 7a in a 2D coordinate frame r=(x,z), with the DoI being the 20×20 black rectangle. For simplicity we normalize all space-units such that the background wave-speed is $v_{0}=1$. Note that the contrast is relatively large with values of $O_{\max}=\pm 0.44$. Note that this example is one of those treated in [37] (see Figure 6, but here the processing is done in the multi-frequency domain as outlined above.

The medium is dominated by stratification along the *z* direction, hence its *K*-space distribution is localized near the $K_{z}$ axis (see discussion below). Referring to the discussion in Section 2.4, it can be recovered using UWB reflection data on the $z_{1}$ plane. We therefore use illumination by a *z*-propagating time-harmonic PW with frequencies in the band $\Omega =\left[0.1,1\right]$. The exact data is generated using method of moments (MoM) simulations. We record only the reflected data over an array of receivers located at z=−150 with |x|<250 with inter-element spacing d=1.15π (larger than the Nyquist distance).

We set b=50, such that the beams are collimated inside the DoI, while maintaining $k_{\min}b\gg 1$ as required for collimation after (23). Using also $k_{\max}=1$ and $\nu_{\max}=1/3$ we obtain from (24) $\left(\bar{x},\;\bar{\xi })=(9.71,\;0.194\right)$. The resulting BF propagators $\left\{{\hat{\Psi }}_{\mu }^{\pm }\left(r\right),\;{\hat{\Phi }}_{\mu }^{\pm }\left(r\right)\right\}$ are calculated via (Equations (C1)–(C5) [21]).

**Figure 8:**
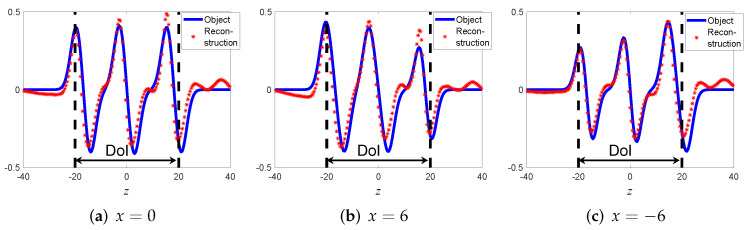
Example A: Comparison of the results along cross sectional cuts parallel to the *z*-axis.

Next we calculate the beam-domain data ${\hat{A}}_{\mu }^{j}$ via (30). The reconstructed object inside the DoI is found using the reflected data imaging field ${\hat{I}}_{1}\left(\omega \right)$ of (31) where we consider only backpropagated beams whose μ axis passes inside the DoI, and then integrating over all frequencies as in (12). The reconstructed medium is illustrated in Figure 7b. As can be seen, the reconstructed object matches well with the object inside the DoI. To better quantify the image results, in Figure 8 we plot cross-sectional cuts of the object at x=0 and at x±6.

**Figure 9:**
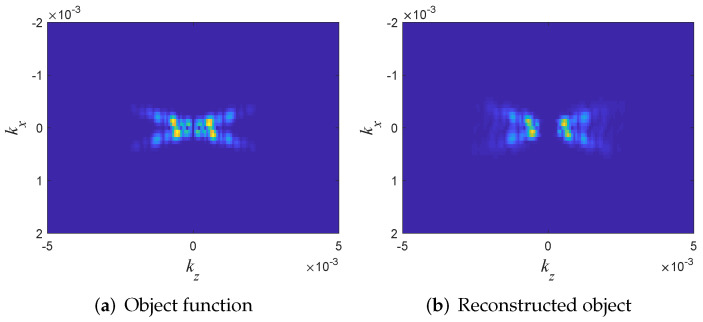
Example A: Comparison of the K-space distributions of the original (**a**) and reconstructed (**b**) objects.

The sources of error are readily seen in the K-space distribution of the original and reconstructed media in Figure 9. Note that the imaging algorithm has recovered most of the object’s K-space, except for the region around |K|≈0. As discussed in Section 2.4, this missing data is due to the low frequency cutoff $k_{min}=0.1$ in the data. The main drawback of the “UWB reflection mode inversion” schemes is the missing transversal spectrum components and the |K|→0 components, (which are small in this example). In general, one may try to recover this data by using transition mode data $\left(z_{2}\right)$ but in many applications this data is not available. Alternatively, one may use several illumination directions which are synthesized from the array data via the method outlined in Phase I of Section 5. These additional illuminations are also used to reduce the reconstruction noise, as explored in II-Section 6 in [37]. The readers are referred to other examples in [36,37,42,43,44,50].

### 5.3. Example B: An Object with Sharp Boundaries. Reflection and Transmission Data

**Figure 10:**
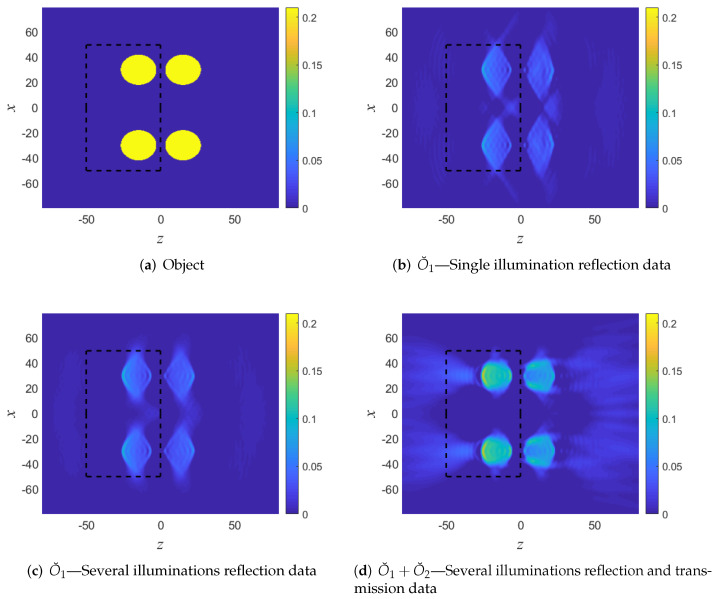
Numerical example B. (**a**) The object function. (**b**) Reconstruction using single illumination and reflection data. (**c**) Reconstruction using several illuminations and reflection data. (**d**) Reconstruction using several illuminations and reflection and transmission data. The DoI is illustrated in a black rectangle.

The object shown in Figure 10a has sharp boundaries, strong transversal *K* components, and a non-zero average (i.e., $\bar{O}(|K|=0)\neq 0$). As before, we consider a 2D problem with r=(x,z) and normalize the space-units such that the background wave-speed is $v_{0}=1$.

The source array is located on the $z_{1}=-150$ plane over |x|<250, with inter-element spacing d=1.15π. Using this array, we may synthesize PW illumination over a spectral range of $\pm 60^{\circ }$ (see discussion in Section 5, Phase I(1–4)). The frequency band is $\Omega =\left[0.1,1\right]$. We consider both the reflection and transmission data over similar receiver arrays at $z_{1}=-150$ and $z_{2}=150$. The exact scattered data is calculated via the MoM.

For the beam processing we use b=20, such that the beams are collimated inside the DoI, while maintaining $k_{\min}b\gg 1$ as required for collimation after (23). Using also $k_{\max}=1$ and $\nu_{\max}=1/3$ we obtain from (24) $\left(\bar{x},\;\bar{\xi })=(6.47,\;0.32\right)$. The resulting BF propagators $\left\{{\hat{\Psi }}_{\mu }^{\pm }\left(r\right),\;{\hat{\Phi }}_{\mu }^{\pm }\left(r\right)\right\}$ are calculated via (Equations (C1)–(C5) [21]).

Figure 10b depicts the reconstructed objects in the front (left) using a single PW illumination at $\theta_{i}=0$ and reflection data at the $z_{1}$ plane. As expected, the reflection data provides good longitudinal resolution but poor transversal resolution (see Figure 2b). Note also that the value of the reconstructed object function is approximately one half of the true value due to the missing data at |K|=0. As expected, the reconstruction of the object outside the DoI is poor.

In Figure 10c we improved the resolution by using several illumination directions (as one may expect by considering Figure 2a,b), yet the reconstruction still suffers from poor transversal resolution and low value of the reconstructed object. These problems are mitigated in Figure 10d where we used both the reflection and transmission data as in (13). Further improvement can be made via iterative schemes [36,37,42,43,44,50].

**Figure 11:**
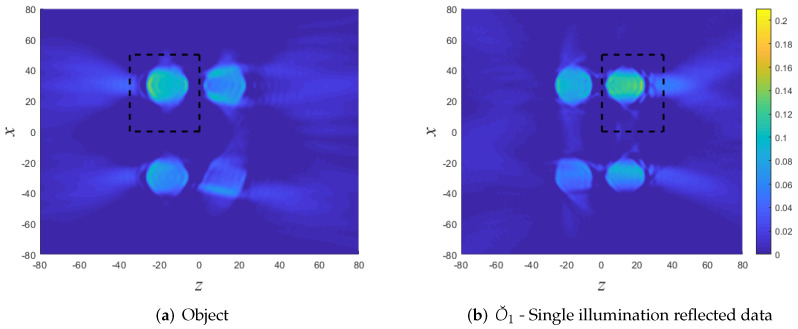
Numerical example B: Local reconstruction in different DoI’s (black rectangles). (**a**) Reconstruction of a cylinder at the front using both reflection and transmission data due to illumination at $\theta_{i}=0$. (**b**) Reconstruction of a cylinder in the back using both reflection and transmission data from several illumination directions.

Finally, in Figure 11 we demonstrate local imaging within different DoIs. Figure 11a depicts the reconstruction of a cylinder at the front (left-top) using both reflection and transmission data due to illumination at $\theta_{i}=0$. The reconstruction is a bit poorer than in Figure 10d where we used several illumination directions. As expected, the reconstruction outside this DoI is poor.

Figure 11b depicts the reconstruction of a cylinder at the back (right-top). Since this cylinder is poorly illuminated by normal incidence, we use here reflection and transmission data from several illumination directions $\theta^{i}=\mp 30^{\circ },\mp 40^{\circ }$. The reconstruction inside the DoI is much better than in Figure 10. As before, the reconstruction outside this DoI is poor.

## 6. Discussion and Conclusions

In this paper, we reviewed the local diffraction–tomography inversion scheme introduced originally in [36,37]. The method is based on a local transformation of the scattering data and local reconstruction using beam backpropagation. It is structured on the concept of beam-frames (BFs). The BFs are a phase–space set of beam-waves that constitute local basis functions (frames) over the propagation domain. As such, they provide an alternative local basis for the global PW or Green’s function radiation integrals. We use the BFs to formulate a local inversion algorithm as an alternative to the conventional approaches. In this and other publications, we demonstrated and explored the advantages of the local algorithm over the conventional PW and Green’s function DT algorithms:

1. Local imaging within a given domain of interest (DoI).
2. Reduced complexity since it accounts only for the beam basis-functions that cover the DoI.
3. Reduced noise level since data and noise arriving from other regions are a priori filtered out.
4. Backpropagation and imaging over a non-homogeneous background.

In previous publications [36,37] we have emphasized TD data processing, where the beam waves are localized space–time wave-packets. This requires somewhat sophisticated mathematics to construct the wave-packets and use them as signal processing tools. The main advantage of the TD approach is the data localization and interpretation. In the present paper, on the other hand, we utilized FD processing followed by an integration over the relevant frequency band. The motivation has been to provide the reader with more straightforward Fourier-based data-processing tools. We also provide the processing tools and closed-form expressions for the local imaging formula, as well as step-by-step guidelines for choosing the various scheme parameters. The method provides an efficient UWB formulation where one has to calculate the beam lattice and propagators only once and then use them for all the frequencies.

## Data Availability

Data associated with this research are available and can be obtained by contacting the author.
